# Green and Economical Synthesis of Bi_2_WO_6_-Doped Geopolymers and Their Visible-Light Catalytic Degradation Mechanism Toward Tetracycline Hydrochloride

**DOI:** 10.3390/ma19153251

**Published:** 2026-08-01

**Authors:** Rui Wang, Wensheng Zhang, Jiayuan Ye, Xianquan Wang, Xuguang Zhou

**Affiliations:** 1 State Key Laboratory of Green Building Materials, China Building Materials Academy, Beijing 100024, China; 2 School of Civil Engineering, NingboTech University, Ningbo 315100, China; 3 Zhejiang Provincial Erjian Construction Group Ltd., Ningbo 315100, China; 4 Qian Xuesen Collaborative Research Center of Astrochemistry and Space Life Science, Institute of Drug Discovery Technology, Ningbo 315211, China

**Keywords:** geopolymers, Bi_2_WO_6_, tetracycline hydrochloride, photocatalytic degradation, mechanism

## Abstract

To address the environmental threats posed by antibiotic residues in water and the engineering bottlenecks of traditional powder photocatalysts—such as high cost, complicated preparation processes—a novel Bi_2_WO_6_-doped geopolymer composite photocatalytic material was successfully constructed through a facile and green synthesis strategy, utilizing low-cost and readily available geopolymers as the supporting matrix. The adsorption and degradation performance of this material toward tetracycline hydrochloride (TC), a typical antibiotic in aquatic environments, was systematically evaluated under visible-light irradiation. Experimental results demonstrate that the composite system exhibits a synergistic effect of adsorption enrichment by geopolymers and in situ photodegradation by Bi_2_WO_6_. Under the optimized conditions of merely 4% Bi_2_WO_6_ loading, catalyst dosage of 1 g/L, and an initial pH of 8.0, the degradation efficiency of TC reached 82.7% after 300 min of visible-light illumination. Even in real and complex water matrices, the material maintained a stable microstructure and highly efficient targeted removal capability. Radical scavenging experiments confirmed that the highly oxidative photogenerated holes (h^+^) generated by Bi_2_WO_6_ and the superoxide radicals (O^2−^) derived from the reduction of dissolved oxygen are the primary active species governing the efficient decomposition of TC. This study not only ameliorates the agglomeration tendency of pure-phase Bi_2_WO_6_ but also, by virtue of its inexpensive raw materials, facile synthesis process, and excellent adaptability to the weakly alkaline environments of actual water sources, provides a highly feasible strategy for the future low-cost, large-scale remediation of realistic aquatic environments and the treatment of industrial antibiotic wastewater.

## 1. Introduction

With the rapid development of intensive animal husbandry and the pharmaceutical and chemical industries, the “pseudo-persistent” residue of antibiotics in aquatic environments has emerged as an ecological focal point of global concern. As a class of broad-spectrum antibiotics with the highest consumption and widest application, tetracyclines (TCs) are extensively utilized in human medical treatment and as growth-promoting additives in livestock farming. However, pharmacokinetic studies reveal that the metabolic rate of TCs in vivo is extremely low. The vast majority of the ingested dosage cannot be fully absorbed and metabolized, ultimately being discharged into the water environment as parent compounds or active metabolites via excreta [1,2]. These residual TCs not only threaten human health through biomagnification within the food chain but also readily induce the proliferation of antibiotic resistance genes (ARGs) in the environment, precipitating the emergence of “superbugs” and posing a protracted threat to aquatic ecosystems [3]. Consequently, the development of highly efficient, economical, and eco-friendly remediation technologies for TCs is of imminent concern for aquatic ecosystems.

At present, the principal strategies for eliminating TCs from aquatic environments encompass biodegradation, adsorption, and advanced oxidation processes (AOPs) [4]. Given the intrinsic and potent antibacterial properties of TCs, conventional biological treatments frequently suffer from low efficiency due to the severe inhibition of microbial activity [5,6]. Meanwhile, although adsorption boasts operational simplicity, it merely facilitates the phase transfer of pollutants without achieving genuine degradation [7]. In comparison, semiconductor-based photocatalytic technology is widely regarded as one of the most promising treatment alternatives owing to its capability to harness solar energy for the generation of highly reactive radicals (e.g., ·OH and·O_2_^−^), which can effectively degrade organic pollutants and partially mineralize them, with the final products being mainly H_2_O and CO_2_ [8,9,10]. Among a plethora of photocatalysts, bismuth tungstate (Bi_2_WO_6_) exhibits tremendous potential in the realm of visible-light-driven antibiotic degradation by virtue of its unique layered structure, suitable bandgap, and exceptional chemical stability [1,11]. Nevertheless, the precursor materials for conventional powder Bi_2_WO_6_ are relatively expensive, and traditional synthetic routes for highly active nanophases (such as high-temperature and high-pressure hydrothermal methods) are energy-intensive and difficult to scale up. More critically, it suffers from the physical defect of being prone to agglomeration and subsequent deactivation in practical applications [12,13,14]. These economic and engineering bottlenecks have severely constrained its industrial-scale implementation in practical, large-scale water treatment scenarios.

To resolve the aforementioned issues, immobilizing photocatalytically active components onto porous frameworks to construct supported catalysts has emerged as a research hotspot. Geopolymers, a class of green inorganic cementitious materials synthesized via the alkali activation of aluminosilicate precursors (e.g., metakaolin and fly ash), are regarded as ideal catalyst supports owing to their three-dimensional network porous structure, superior mechanical strength, and low preparation cost [15]. Their unique porous frameworks not only serve as excellent dispersion media for Bi_2_WO_6_, effectively suppressing the agglomeration of active components and thereby exposing more photocatalytic active sites, but also enrich TCs in water via physical adsorption, significantly accelerating the degradation kinetics through an “adsorption–photocatalysis” synergistic effect. Distinct from the complicated dispersion–suspension solidification processes conventionally employed in the literature to fabricate geopolymer microspheres [16], this study endeavors to explore a more economical and facile “one-step” doping and molding route to accommodate the demands of actual large-scale wastewater treatment. Furthermore, considering that actual industrial wastewater and natural water bodies frequently exhibit weak alkalinity, the development of photocatalytic materials that maintain high efficiency and stability in slightly alkaline environments is of significant practical importance.

Based on the aforementioned considerations, this study proposes a low-cost and easily scalable strategy for constructing composite catalysts. Initially, the photocatalytically active phase, Bi_2_WO_6_, was prepared via a facile solid-state oxide calcination method. Subsequently, it was uniformly immobilized onto the green and low-carbon mineral geopolymer (GMs) matrix through a simple mechanical stirring–blending and room temperature alkali activation curing process, successfully constructing Bi_2_WO_6_@GMs composites with various doping ratios. Utilizing characterization techniques such as XRD, FTIR, SEM, BET, and UV-Vis DRS, the spatial distribution patterns of the active components within the mesoporous matrix and the evolution of the microstructures were profoundly elucidated. This work not only systematically evaluated the highly efficient “enrichment–adsorption and photocatalysis” synergistic degradation performance of the material toward TC but also broke through the limitations of traditional ideal pure water systems by emphatically investigating its anti-interference capability in complex and realistic water matrices, such as tap water and river water. The intrinsic mechanisms by which the composite maintains superior catalytic activity under naturally weakly alkaline environments (pH ≈ 8) without the need for additional pH adjustment were explored. Furthermore, combined with radical scavenging experiments, the core reaction pathways dominated by photogenerated holes (h^+^) and superoxide radicals (O_2_^−^) generated from the reduction of dissolved oxygen were clarified. This study not only successfully developed a practical functional material for water treatment that is green, economical, easy to process, and highly adaptable to actual environments but also provided a theoretical basis and engineering technical support of significant reference value for the large-scale in situ purification of antibiotic wastewater and natural water bodies.

## 2. Experiment

### 2.1. Materials and Reagents

Tungsten trioxide (reagent grade, 99%), bismuth oxide (reagent grade), sodium hydroxide (95%), tetracycline hydrochloride (biotech grade), p-benzoquinone (97%), methanol (99.5%), and isopropanol (99.5%) were purchased from Shanghai Macklin Biochemical Co., Ltd. (Shanghai, China). Hydrochloric acid (36.5%) was obtained from Guangzhou Hewei Pharmaceutical Technology Co., Ltd. (Guangdong, China). Metakaolin (97%) was supplied by Inner Mongolia Super Building Material Technology Co., Ltd. (Hohhot, China). Sodium silicate solution (water glass, 35.84%) was acquired from Jiashan Yourui Refractory Co., Ltd. (Jiaxing, China).

### 2.2. Sample Synthesis

#### 2.2.1. Synthesis of Bi_2_wo_6_

The Bi_2_WO_6_ photocatalytic powder was synthesized via a solid-state reaction method. In accordance with the stoichiometric ratio of n(Bi_2_O_3_): n(WO_3_) = 1:1, bismuth oxide and tungsten trioxide were accurately weighed at a mass ratio of 2:1 as the raw materials. The mixed precursors were transferred into an agate mortar and thoroughly ground into fine powder to ensure compositional homogeneity, after which they were loaded into an alumina crucible, spread evenly, and placed in the center of a muffle furnace. The calcination program was set to ramp to 500 °C at a heating rate of 5 °C·min^−1^, followed by an isothermal hold at this temperature for 4 h. Upon completion of the reaction, the sample was allowed to cool naturally within the furnace to below 150 °C before retrieval. Finally, the obtained product was reground and passed through a 200–400 mesh sieve to acquire the Bi_2_WO_6_ catalyst powder with uniform particle size distribution.

#### 2.2.2. Synthesis of Bi_2_WO_6_@GMs Composites

Geopolymer-based composites with varying Bi_2_WO_6_ contents were prepared via a direct doping method. Initially, the alkali activator was formulated by adding solid NaOH into a sodium silicate solution with a modulus of 3.3. Through continuous stirring until the complete dissolution of NaOH, the modulus of the sodium silicate solution was adjusted to 1.2. Because the dissolution process was accompanied by an intense exothermic reaction, the obtained alkali activator was required to stand and cool for 24 h to ensure performance stability. During sample preparation, the cooled alkali activator was poured into a mixing bowl at an alkali-to-solid ratio of 0.8, followed by the addition of metakaolin and the pre-synthesized Bi_2_WO_6_ powder (with Bi_2_WO_6_ doping levels set at 0%, 2%, 4%, and 6% of the total mass, respectively). After 10 min of manual stirring, the mixture was first subjected to low-speed agitation to ensure thorough wetting of the materials and then switched to high-speed stirring until a homogeneous slurry was formed. The slurry was poured into molds coated with a release agent and properly labeled. A vibrating table was employed to eliminate entrapped air bubbles, after which the molds were covered with plastic wrap and cured at room temperature for 1 day. Following initial setting, the samples were transferred into an oven at 60 °C for thermal curing for 16 h. Subsequently, they were demolded, rewound with plastic wrap, and continuously cured at 60 °C up to 48 h to obtain the final Bi_2_WO_6_@GMs composites.

### 2.3. Characterization Methods

Various analytical techniques were employed to analyze the physicochemical properties of the Bi_2_WO_6_@GMs composites. The crystalline phase structures of Bi_2_WO_6_@GMs before and after the degradation of tetracycline hydrochloride were characterized using an X-ray powder diffractometer (XRD, D8 Advance, Bruker, Ettlingen, Germany). Fourier transform infrared spectroscopy (FTIR, Nicolet iS 5, Thermo Fisher Scientific, Waltham, MA, USA) was utilized to determine the chemical bonds and functional group information of Bi_2_WO_6_@GMs before and after the reaction. The microscopic morphology and elemental distribution of Bi_2_WO_6_@GMs were observed via a scanning electron microscope (SEM, Quattro S, Thermo Fisher Scientific, Waltham, MA, USA). The specific surface area was measured utilizing a surface area and porosity analyzer (BET, ASAP 2460, Micromeritics (Shanghai, China) Instrument Co., Ltd., Shanghai, China). Furthermore, the optical absorption properties of Bi_2_WO_6_@GMs were analyzed employing a UV-Vis diffuse reflectance spectrophotometer (UV-Vis DRS, UV-3600 Plus, Shimadzu Corporation, Kyoto, Japan).

### 2.4. Evaluation of Photocatalytic Activity

The photocatalytic experiments employed a 300 W xenon lamp (PL-X300, Beijing Perfectlight Technology Co., Ltd., Beijing, China) as the light source, operating at a current of 10 A and equipped with a 400 nm cutoff filter to obtain visible light. The typical irradiance at the sample surface is approximately 150 mW/cm^2^. A tetracycline hydrochloride solution of the desired concentration was prepared and stirred in the dark for 20 min to ensure uniform dispersion. A specific mass of Bi_2_WO_6_@GMs was weighed and added into the tetracycline hydrochloride solution, followed by stirring in the dark for 20 min to establish adsorption–desorption equilibrium. Subsequently, the light source was turned on, and, at 30 min intervals, 5 mL of the suspension was sampled and filtered through a 0.45 μm syringe filter. The absorbance of the filtrate at 357 nm [17] was measured utilizing a UV-Vis spectrophotometer (756S, Shanghai Lengguang Technology Co., Ltd., Shanghai, China). The degradation efficiency of the catalyst was calculated according to the following equation:

(1) $$E=\frac{\left(C_{0}-C_{t}\right)}{C_{0}}\times 100\%$$

where *E* represents the photocatalytic degradation efficiency of Bi_2_WO_6_@GMs toward tetracycline hydrochloride,$\;C_{0}$ is the absorbance after dark adsorption equilibrium, and $C_{t}$ is the absorbance at irradiation time t.

## 3. Results and Discussion

### 3.1. Characterization Results

#### 3.1.1. XRD

**Figure 1:**
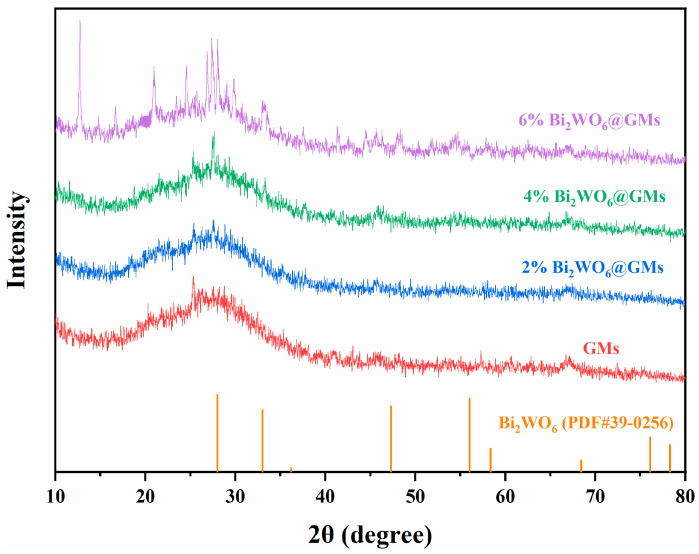
XRD patterns of Bi_2_WO_6_@GMs with varying Bi_2_WO_6_ loading amounts.

Figure 1 illustrates the XRD patterns of Bi_2_WO_6_@GMs with varying Bi_2_WO_6_ loading amounts. All samples exhibit a distinct broad and diffuse hump in the 2θ range of 20–38°, which is attributed to the amorphous geopolymer gel network formed by metakaolin under strong alkali activation. With the increase in Bi_2_WO_6_ doping content, the characteristic diffraction signals corresponding to its orthorhombic standard card (JCPDS No. 39-0256) gradually intensify. Notably, the main diffraction peak (131) located at around 28.3° exhibits an obvious trend of increasing intensity, visually reflecting the variation in the loading amount of the active components within the composites [18]. The linear evolution of the main peak intensity and the stability of the peak position fully corroborate that the Bi_2_WO_6_ particles have been successfully and uniformly integrated into the geopolymer network, maintaining excellent structural integrity of the crystalline phase during the alkali activation and subsequent thermal curing processes. Because the maximum mass fraction of Bi_2_WO_6_ in the composites is merely 6%, its overall diffraction contribution is relatively limited. Consequently, apart from the strongest main diffraction peak, the diffraction signals of other crystal planes are highly susceptible to being submerged in the intense amorphous backscattering background noise of the geopolymer matrix, failing to present distinct peak profiles in the patterns [19].

#### 3.1.2. FTIR

**Figure 2:**
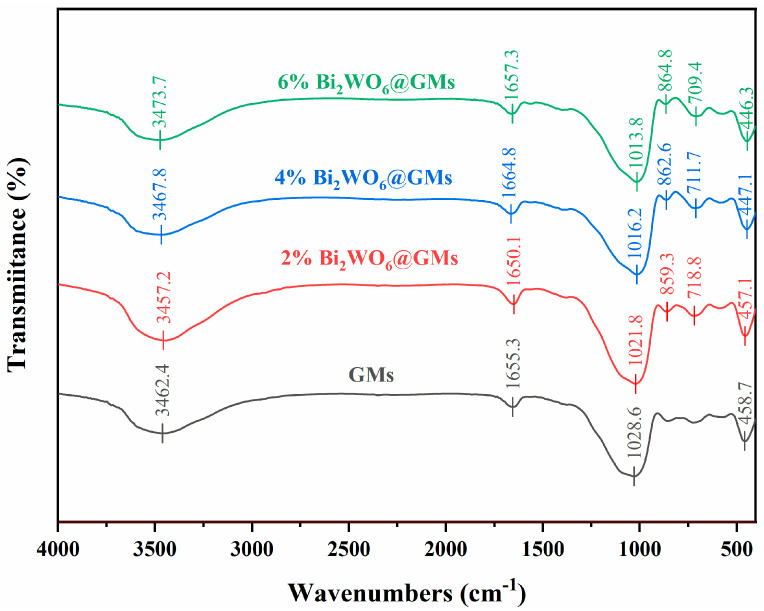
FTIR spectra of Bi_2_WO_6_@GMs with varying Bi_2_WO_6_ loading amounts.

Figure 2 displays the FTIR spectra of Bi_2_WO_6_@GMs with varying Bi_2_WO_6_ loading amounts. All samples exhibit a broad characteristic absorption band around 3460 cm^−1^, which is attributed to the stretching vibrations of adsorbed water and structural hydroxyl groups (-OH) within the pores of the geopolymer gel. Coupled with the H-O-H bending vibration peak appearing around 1650 cm^−1^, this reveals the presence of adsorbed water and chemically bound water within the hydration product gel [20]. The strong absorption band located at around 1020 cm^−1^ is assigned to the asymmetric stretching vibration of Si-O-Al (Si) in the geopolymer network, while the absorption peak around 450 cm^−1^ corresponds to the bending vibration mode of the Si-O-Al (Si) bonds. Both of these jointly signify the successful construction of the three-dimensional aluminosilicate framework [21,22].

With the increase in the Bi_2_WO_6_ loading amount, the composites manifest distinct characteristic absorption peaks at 860 cm^−1^ and 720 cm^−1^. The former corresponds to the symmetric stretching vibration of the W-O bonds in the Bi_2_WO_6_ crystal lattice, whereas the latter is assigned to the vibration mode of the W-O-W bridging oxygen bonds in the layered structure [23]. It is noteworthy that because the characteristic vibration sites of Bi_2_WO_6_ and the background absorption sites of the geopolymer matrix are highly proximal in wavenumber, their signals undergo spectral superposition within the geopolymer network. This synergistic effect results in a significant enhancement of the absorption intensity in this region with increasing doping content, which not only verifies the successful integration of the photocatalytic components but also reflects the excellent physical compatibility between Bi_2_WO_6_ and the dense geopolymer interface.

#### 3.1.3. SEM and BET Analyses

**Figure 3:**
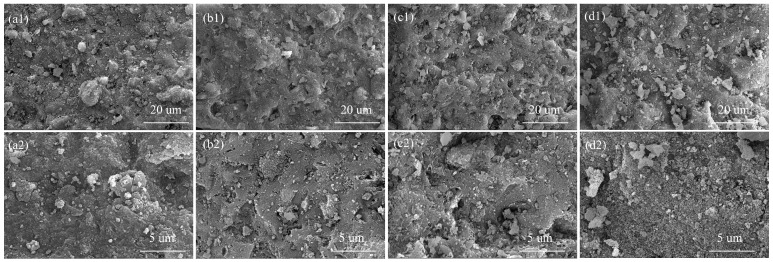
SEM images of Bi_2_WO_6_@GMs with varying Bi_2_WO_6_ loading amounts. (**a1**,**a2**), (**b1**,**b2**), (**c1**,**c2**), and (**d1**,**d2**) represent the pure GMs, 2%, 4%, and 6% groups, respectively.

**Figure 4:**
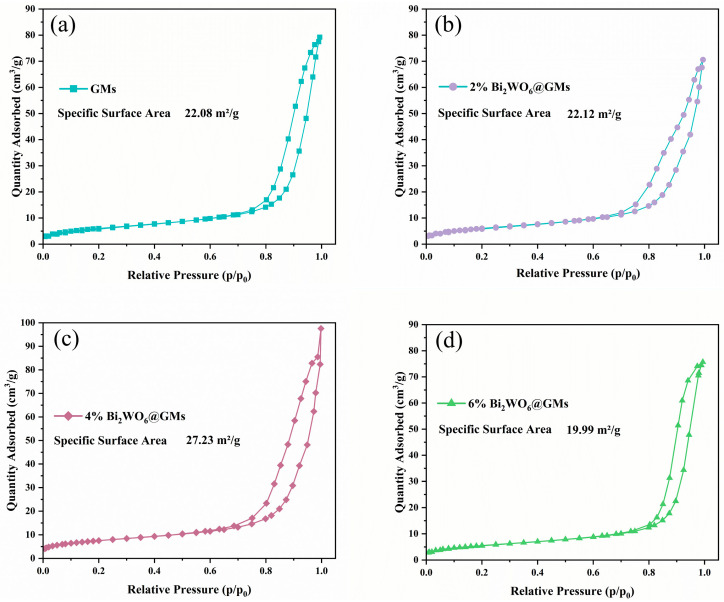
Adsorption–desorption isotherms of Bi_2_WO_6_@GMs with varying Bi_2_WO_6_ loading amounts.

Through the systematic characterization of the microscopic morphology and pore structure of the samples, the SEM images shown in Figure 3 visually reflect the distribution evolution of the Bi_2_WO_6_ particles within the geopolymer matrix. The pure GMs group exhibits the characteristics of a highly continuous and dense N-A-S-H gel, featuring a smooth surface without obvious microcracks or macroscopic voids, which corroborates the excellent structural integrity of the matrix. With the increase in loading amount, the active particles are effectively anchored onto the gel surface or embedded within the framework. Among them, the 4% group sample demonstrates the optimal interfacial compatibility and dispersion uniformity, whereas the 6% group sample presents local particle stacking and agglomeration phenomena. Figure 4 illustrates the N_2_ adsorption–desorption isotherms of the samples. All curves display the characteristics of Type IV isotherms with hysteresis loops, indicating the development of typical mesoporous structures within the composites [24]. Analyzed from the perspective of adsorption performance, although the loading of Bi_2_WO_6_ increases the compositional complexity, constrained by the strong “encapsulation effect” of the geopolymer matrix and its inherently dense framework, the overall specific surface area of the composites remains at a relatively low level (<30 m^2^/g). This, to a certain extent, limits the absolute number of surface active sites.

**Figure 5:**
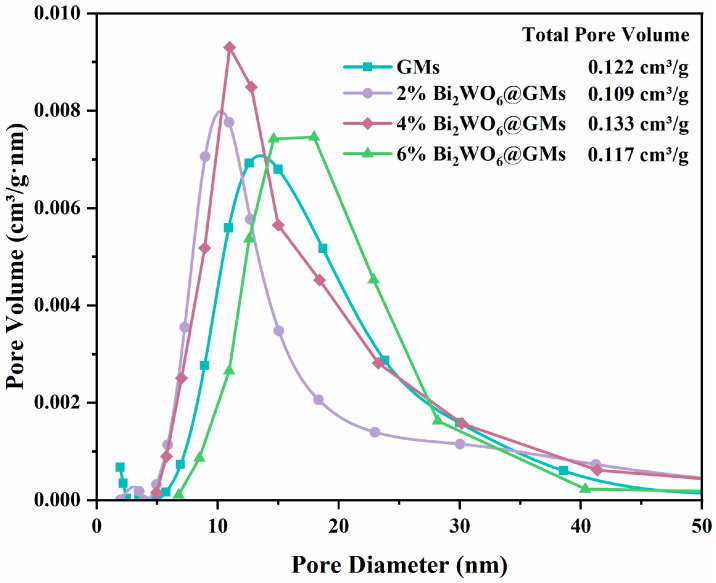
Pore size distribution curves of Bi_2_WO_6_@GMs with varying Bi_2_WO_6_ loading amounts.

However, further combined with the pore size distribution curves in Figure 5, it is evident that the pore sizes of the samples are mainly concentrated in the range of 10–40 nm, a scale significantly larger than the kinetic diameter of the tetracycline hydrochloride molecule (approximately 1.3 nm) [25,26]. Such suitable mesoporous channels drastically reduce the mass transfer resistance for the diffusion of pollutant molecules into the interior of the material, facilitating the rapid establishment of adsorption equilibrium. During the photocatalytic process, the larger pore size not only ensures sufficient contact between the reactants and the active sites but also promotes the timely outward migration of degradation products, preventing the coverage of catalytic sites by intermediates. When the loading amount is elevated to 6%, the physical filling of excessive particles blocks partial effective pore channels, leading to a decline in the peak intensity of the pore size distribution and an increase in diffusion resistance. Such structural alterations may exert a negative impact on its macroscopic photocatalytic degradation efficiency by shielding active sites and hindering proton transfer.

#### 3.1.4. UV-Vis DRS

**Figure 6:**
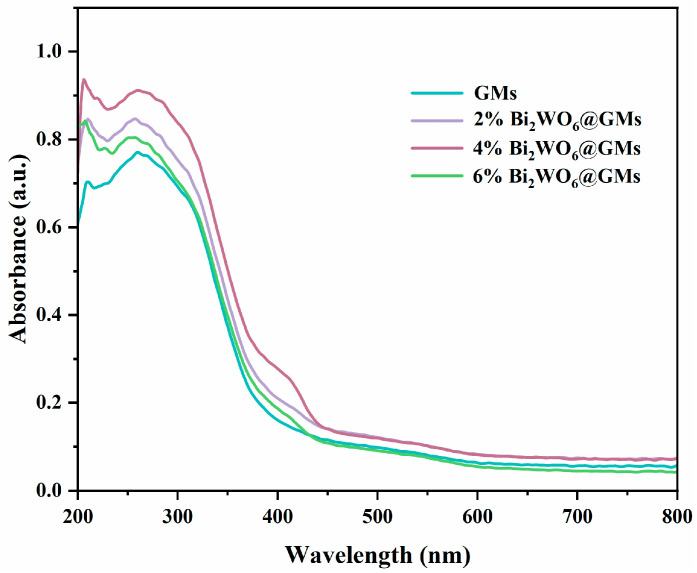
UV-Vis DRS spectra of Bi_2_WO_6_@GMs with varying Bi_2_WO_6_ loading amounts.

Figure 6 displays the UV-Vis DRS spectra of Bi_2_WO_6_@GMs with varying Bi_2_WO_6_ loading amounts. Pure GMs possess virtually no optical absorption capacity in the visible light region. Nevertheless, through the loading of functional components, the composites exhibit a pronounced characteristic absorption edge in the 400–450 nm band, signifying the successful extension of their optical response range from the ultraviolet to the visible light region. With the increase in Bi_2_WO_6_ density, the optical absorption intensity of the composites manifests a distinct gradient enhancement, and the absorption edge is accompanied by a slight red-shift phenomenon. This primarily originates from the highly dispersed integration of the photocatalytically active components within the geopolymer network, alongside the potential electronic coupling effect at their interface. However, when the doping level is elevated to 6%, the optical absorption intensity of the material undergoes a significant decline. Coupled with the analysis of the microscopic morphology of the samples, this may be associated with the agglomeration of Bi_2_WO_6_ particles.

### 3.2. Photocatalytic Results

**Figure 7:**
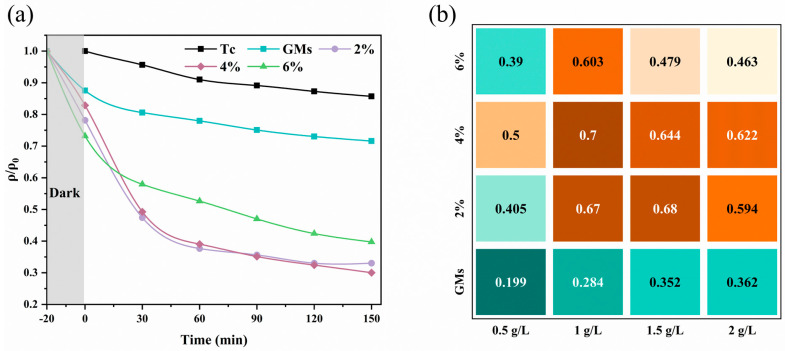
(**a**) Degradation efficiencies of various samples at a dosage of 1 g/L and (**b**) heatmap of degradation efficiencies at varying catalyst dosages. (The color scale represents the degradation efficiency (0 = 0%, 1 = 100%)).

Initially, kinetic experiments were conducted under the conditions of an initial TC concentration of 10 mg/L and a catalyst dosage of 1 g/L, with the results presented in Figure 7a [27,28]. As depicted in the figure, tetracycline hydrochloride undergoes spontaneous decomposition under illumination. However, the efficiency is extremely low. The removal efficiency of pure GMs under visible light irradiation is 28.4%, which primarily originates from mesoporous physical adsorption, as the matrix itself lacks photocatalytic activity. With the introduction of Bi_2_WO_6_, the degradation efficiency of the composites is significantly enhanced. Among them, the sample with 4% loading exhibits the strongest photodegradation kinetic response, achieving a degradation efficiency of 70% after 150 min of illumination. This conclusion is highly consistent with the optimal particle dispersion observed via SEM and the unhindered 10–40 nm mesoporous channels confirmed by BET. Namely, the 4% loading amount ensures sufficient active sites while circumventing the excessive filling of the geopolymer pore channels by particles, thereby achieving an optimal balance between the generation of photogenerated charge carriers and the mass transfer of pollutants. In contrast, the degradation efficiency of the sample with 6% loading exhibits a pronounced decline. This is attributed to the self-shielding effect of active sites caused by particle agglomeration, as well as the light scattering loss resulting from the increased suspension turbidity induced by excessive opaque particles, which collectively limit the penetration depth of visible light into the interior of the catalyst. To comprehensively evaluate the photocatalytic performance of the composites and determine the optimal reaction parameters, Figure 7b presents a two-dimensional heatmap of the loading amount and catalyst dosage. The results reveal that 4% loading combined with a dosage of 1 g/L constitutes the optimal reaction window for this system, fully corroborating the successful synergy between the geopolymer matrix and the Bi_2_WO_6_ active phase. This combination guarantees the sufficient exposure of active sites while minimizing particle agglomeration and light scattering losses to the greatest extent, thereby actualizing the highly efficient conversion of photochemical energy into chemical energy.

Pseudo-first-order and pseudo-second-order kinetic fitting are performed using the degradation data presented in Figure 7a. The fitting results are summarized in Table 1.

According to Table 1, for all Bi_2_WO_6_@GMs composites, the pseudo-second-order model yields significantly higher correlation coefficients than the pseudo-first-order model, indicating that the degradation process is better described by pseudo-second-order kinetics. This indicates that the chemisorption process may be consistent with the proposed adsorption–photocatalysis synergistic mechanism.

Among the samples, the 4% Bi_2_WO_6_@GMs exhibits the highest pseudo-second-order rate constant, which is approximately 1.5 times that of the 2% sample and 1.3 times that of the 6% sample. This trend aligns well with the degradation efficiency results (Figure 7a). The lower $k_{2}$ value for the 2% sample is attributable to insufficient active sites, while the decreased $k_{2}$ for the 6% sample likely results from particle agglomeration and pore blockage, which reduce the accessible surface area and hinder the diffusion of TC molecules to the active sites.

Overall, the kinetic analysis confirms that the 4% Bi_2_WO_6_@GMs composite possesses the optimal photocatalytic activity, and the chemisorption-controlled process supports the crucial role of surface adsorption in the overall degradation mechanism.

**Figure 8:**
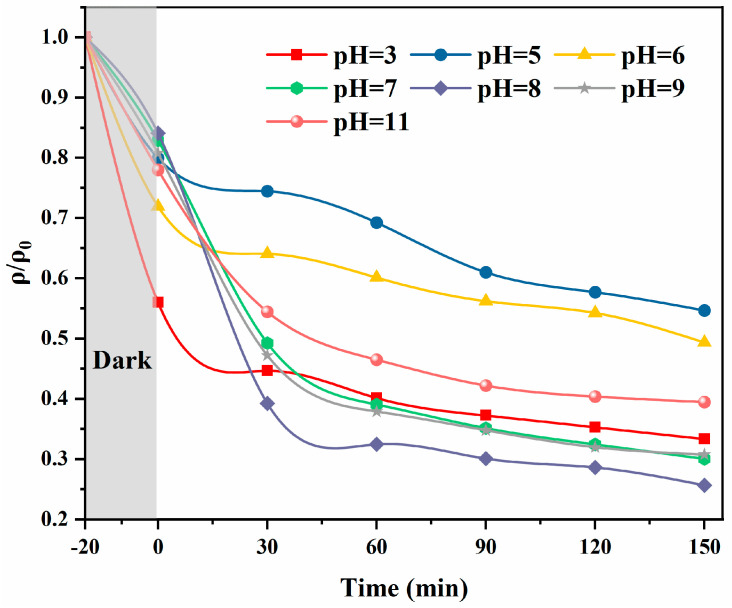
Degradation efficiencies of 4% Bi_2_WO_6_@GMs at various pH values.

After determining the optimal ratio of the loading amount (4%) and the solid-to-liquid ratio (1 g/L), it is necessary to further investigate the effect of the initial pH value of the reaction system on the degradation efficiency. Figure 8 presents the experimental results. In the pH range of 5–11, the degradation efficiency exhibits a trend of initially increasing and subsequently decreasing, reaching a peak value of 74.3% at a pH of 8. This pattern reflects the synergistic regulatory effect of the solution’s acidity and alkalinity on the surface charge of the catalyst and the chemical speciation of the pollutant molecules. When the pH is around 8, the system reaches the optimal matching point between the generation rate of reactive radicals and the adsorption equilibrium of the pollutant molecules. Because the GMs framework is negatively charged, whereas TC molecules are predominantly in a neutral or weakly anionic state at pH = 8, the electrostatic environment at this point is most conducive to the migration of reactive species and their contact with the reaction interface. It is noteworthy that under the acidic condition with an initial pH of 3, the system also exhibits a relatively high removal efficiency, which is primarily attributed to the unique chemical stability and alkaline buffering capacity of the geopolymer material. The synthesis process of the GMs matrix involves strong alkali activation, endowing its internal structure with a high intrinsic alkalinity. Only when the initial pH is set to 3 can the acidic environment neutralize part of the alkalinity released by the matrix, enabling the reaction system to maintain an acidic state. According to the dissociation characteristics of TC, TC molecules mainly exist in a cationic state (TCH_3_^+^) in an acidic environment, generating a strong electrostatic adsorption interaction with the negatively charged GMs framework ([AlO_4_]^−^ units), thereby significantly enhancing the adsorption and capture capacity and improving the overall removal efficiency.

Considering that most industrial wastewater and natural water sources typically exhibit slightly alkaline characteristics (pH 7–9), the optimal reaction window of the 4% Bi_2_WO_6_@GMs system is highly compatible with real-world aqueous environments. This implies that in practical engineering applications, the material eliminates the necessity for complex pre-neutralization and pH adjustment of the wastewater, which not only significantly simplifies the operational procedures but also reduces chemical reagent consumption and associated treatment costs. Such outstanding adaptability to near-neutral and weakly alkaline environments fully corroborates the broad application prospects of this geopolymer-based composite in the field of low-carbon and cost-effective water treatment.

**Figure 9:**
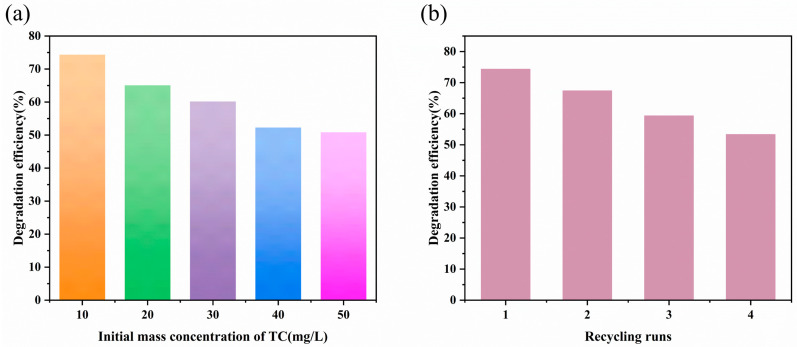
Degradation efficiencies of 4% Bi_2_WO_6_@GMs at varying tetracycline hydrochloride concentrations and over consecutive reuse cycles. (**a**) Degradation efficiency at different concentrations, (**b**) Degradation efficiencies at consecutive reuse cycles.

Based on the determination of the optimal process parameters, including a 4% loading amount, a 1 g/L solid-to-liquid ratio, and an initial pH of 8, this study further investigated the effects of initial concentration and cyclic stability on the degradation performance. As illustrated in Figure 9a, with the initial concentration of tetracycline hydrochloride increasing from 10 mg/L to 50 mg/L, the degradation efficiency after 150 min of reaction gradually decreases from 74.3% to 50.8%. This trend is attributed to “site competition” in the photocatalytic reaction. Under the premise of a fixed catalyst dosage, high-concentration pollutant molecules rapidly occupy the limited active sites, leading to adsorption saturation and consequently a reduction in efficiency per unit degradation load. The cyclic stability test of the material is presented in Figure 9b. After undergoing four consecutive degradation cycles, the degradation efficiency progressively drops to 53.3%. The step-like attenuation of the degradation performance is primarily ascribed to the cumulative residue of reaction intermediates on the surface of Bi_2_WO_6_ (possible site blocking by residual intermediates), which gradually shields part of the effective reaction sites. Nevertheless, the system still maintains a degradation capacity of over 50% after multiple cycles.

**Figure 10:**
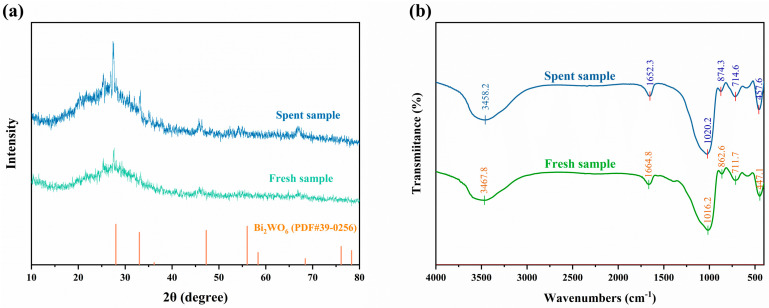
Comparison of (**a**) XRD patterns and (**b**) FTIR spectra of 4% Bi_2_WO_6_@GMs before and after the reaction.

Subsequent characterization analysis was performed on the post-experiment materials, with the results illustrated in Figure 10. The XRD results demonstrate that the diffraction pattern of the sample after the reaction is fundamentally consistent with that before the reaction. It still maintains the typical amorphous diffuse hump characteristic in the 20–38° range. Simultaneously, the main diffraction peak position of Bi_2_WO_6_ does not exhibit any obvious shift, and no new diffraction peaks emerge. This indicates that neither crystalline phase transition nor structural destruction occurs within the composite during the photocatalytic reaction, and both the crystal structure of Bi_2_WO_6_ and its dispersion state in the GMs remain stable. The FTIR spectra further corroborate this result. The positions of the characteristic absorption peaks of the samples before and after the reaction, such as those at 3460 cm^−1^ (–OH stretching vibration), 1650 cm^−1^ (H–O–H bending vibration), 1020 cm^−1^ (asymmetric stretching vibration of Si–O–Al(Si)), and 450 cm^−1^ (bending vibration of Si–O–Al(Si)), are basically identical, with no new absorption peaks or obvious peak shifts observed. Meanwhile, the characteristic vibration peaks of W–O and W–O–W assigned to Bi_2_WO_6_ at 860 cm^−1^ and 720 cm^−1^ remain clearly discernible, indicating that the photocatalytically active components undergo neither structural alteration nor leaching during the reaction process. The above results reveal that both the phase composition and chemical structure of the Bi_2_WO_6_@GMs composites remain stable before and after the reaction, manifesting excellent structural stability and interfacial bonding stability. In the cyclic experiments, the primary mechanism for the decline in degradation capacity can be attributed to the gradual saturation of the mesoporous adsorption sites of the GMs. However, in the dynamic continuous-flow environment of actual large-scale water treatment, the enhanced aqueous phase movement and mass diffusion processes are expected to significantly alleviate this interfacial saturation limitation, thereby enabling this composite system to maintain more durable and highly efficient degradation vitality in practical applications.

**Figure 11:**
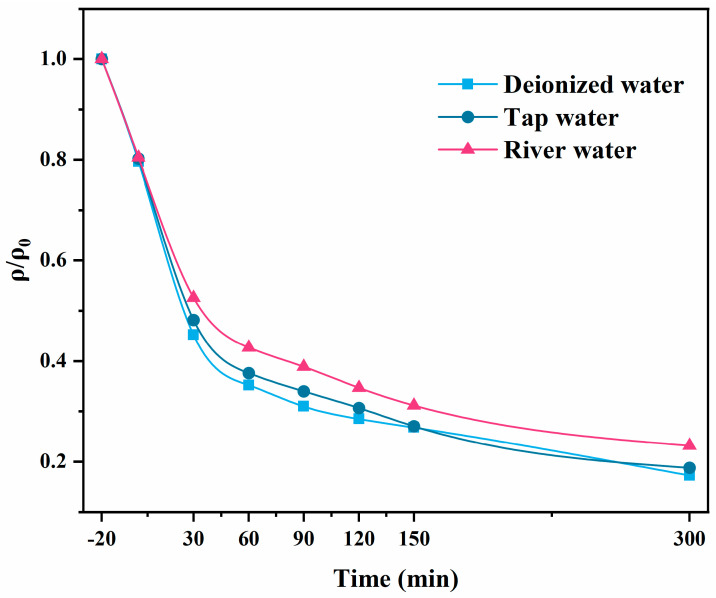
Degradation efficiencies of 4% Bi_2_WO_6_@GMs in various water sources.

Considering the potential interference of complex real-world aqueous environments with the catalytic system, this study systematically investigated the effects of deionized water (pH 7.8), tap water (pH 8.2), and river water (pH 8.5) on the degradation efficiency of tetracycline hydrochloride, thereby further evaluating the practical application potential of the Bi_2_WO_6_@GMs composites. As can be seen from the experimental results shown in Figure 11, all systems exhibit a significant degradation trend over a prolonged reaction period of 300 min, although the degradation efficiencies present slight variations owing to the differences in water matrices. First, in the deionized water system, due to the absence of interference from impurity ions, the material demonstrates the optimal kinetic response and the highest degradation efficiency. TC molecules can achieve maximum effective contact with photogenerated radicals, reaching a degradation rate of 82.7% after 300 min of illumination. Second, in the tap water system, the degradation rate is scarcely affected, yielding a final degradation efficiency of 81.2%. Finally, in the river water system, which contains the most complex environmental components, a slight inhibition phenomenon occurs, resulting in a final degradation efficiency of 77.8%. This is because the natural organic matter (NOM) present in the river water not only undergoes competitive adsorption with TC molecules but may also competitively absorb a portion of the incident light, creating a certain shielding effect and thereby reducing the utilization rate of photogenerated charge carriers per unit time. It is noteworthy that despite the influence of the complex water matrices, 4% Bi_2_WO_6_@GMs still maintains relatively high removal efficiencies across all three water sources at 300 min, without any obvious signs of catalyst poisoning or deactivation. This indicates that it maintains robust performance even under complex ion interference.

To comprehensively evaluate the practical application potential of the Bi_2_WO_6_@GMs composites prepared in this study, we systematically compared them with other advanced Bi_2_WO_6_-based photocatalysts reported in recent years in terms of synthesis parameters and degradation performance, as shown in Table 2. First, regarding the synthesis process, current mainstream high-efficiency composite materials generally rely on liquid-phase hydrothermal or solvothermal methods in specialized high-pressure autoclaves. Such stringent conditions severely limit their large-scale preparation. In contrast, the atmospheric pressure high-temperature calcination process adopted in this study is facile to operate, requires no complex equipment, and possesses remarkable potential for large-scale industrial scale-up. Second, in terms of raw material costs, reducing the absolute usage of expensive semiconductors is the key to advancing towards practical engineering applications. However, the vast majority of systems reported in the literature still utilize expensive photocatalytically active components as the predominant constituent, with the mass fraction of Bi_2_WO_6_ (or the Bi element) in these composites often reaching over 90%. In stark contrast, this study ingeniously utilizes the excellent dispersion and loading capacity of porous geopolymers, ensuring that the mass fraction of Bi_2_WO_6_, which plays the core catalytic role in the formed microspheres, is a mere 4%, while the remaining 96% consists of ultra-low-cost geopolymer carriers. Therefore, the raw material cost is drastically reduced. This strategy of a “ low-cost carrier and ultra-low loading amount” not only simplifies the preparation process but also substantially reduces the material cost, demonstrating considerable commercial and environmental engineering application value.

### 3.3. Photocatalytic

To gain deep insight into the core photocatalytic mechanism of the Bi_2_WO_6_@GMs composites for tetracycline hydrochloride degradation, this study introduced methanol, p-benzoquinone (BQ), and isopropanol (IPA) as targeted scavengers for photogenerated holes (h^+^), superoxide radicals (O_2_^−^), and hydroxyl radicals (·OH), respectively, to identify the primary reactive species in the reaction system [39].

**Figure 12:**
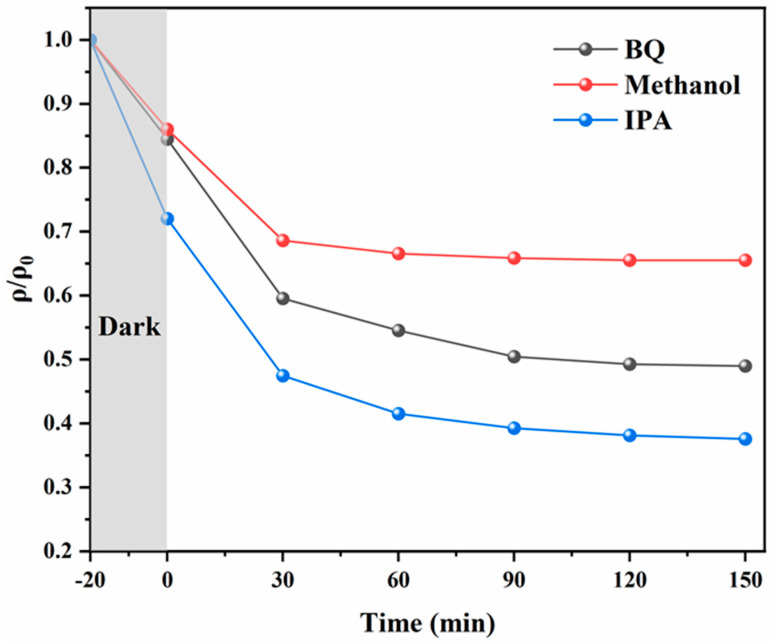
Photocatalytic degradation of TC over 4% Bi_2_WO_6_@GMs in the presence of various radical scavengers.

**Figure 13:**
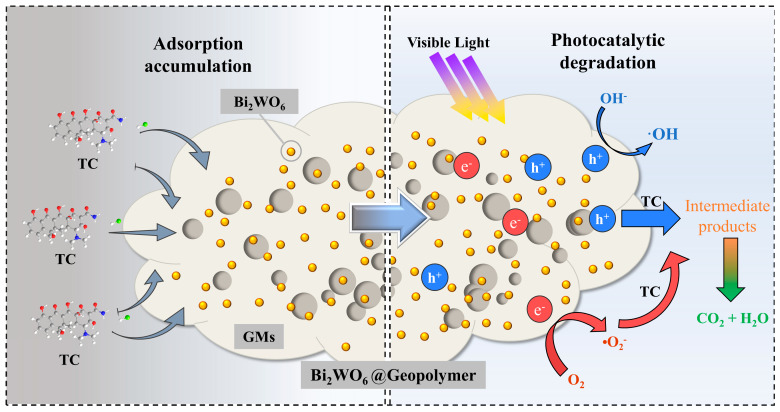
Adsorption–photocatalytic degradation mechanism of tetracycline hydrochloride by Bi_2_WO_6_@GMs.

The results of the scavenging experiments are illustrated in Figure 12. The addition of scavengers led to a decline in the degradation rate of TC by the composite, indicating that the degradation of TC is a result of reactive species participating in the photo-oxidation process. Specifically, after adding methanol, the degradation rate of the system dropped sharply, showing the most intense inhibitory effect. This strongly proves that photogenerated holes (h^+^) occupy an absolute core and dominant position in the entire photocatalytic oxidation reaction. This phenomenon is highly consistent with the unique energy band structure of Bi_2_WO_6_, which possesses an extremely deep (highly positive) valence band potential (approximately +3.0 eV). This endows its photogenerated holes with exceptionally efficient direct oxidation capability, enabling them to directly abstract electrons from TC molecules adsorbed on the catalyst surface and destroy their structures [40,41]. Meanwhile, the addition of BQ also exerted a significant inhibitory effect on the degradation process, confirming the effective generation and synergistic participation of O_2_^−^ within the system. The generation of·O_2_^−^ can be attributed to local defects or oxygen vacancies (O_v_) on the composite surface acting as electron capture centers, which not only effectively suppress the recombination of photogenerated charge carriers but also alter the local reduction potential, facilitating the single electron reduction of dissolved oxygen [42,43,44]. In contrast, the change in the system’s degradation rate after adding IPA was not significant, indicating that the contribution of O_2_^−^ in this system is negligible. Ultimately, the adsorption–photocatalytic degradation mechanism of TC by Bi_2_WO_6_@GMs is depicted in Figure 13. Relying on the strongly oxidizing h^+^ and O_2_^−^ generated via light excitation, a dominant synergistic oxidation occurs on the well-developed mesoporous interface of the geopolymer against the target TC molecules, gradually leading to ring opening, bond cleavage, and eventual decomposition into CO_2_ and H_2_O. Based on the above mechanistic analysis, the core chemical equations of the reaction system are as follows:

(2) $${Bi}_{2}{WO}_{6}+h\nu \to {Bi}_{2}{WO}_{6}\left(e^{-}+h^{+}\right)$$

(3) $$Oᵥ+e^{-}\to e^{-}\left(trapped\right)$$

(4) $$e^{-}\left(trapped\right)+O^{2}\to \cdot \;O^{2-}$$

(5) $$TC+{\;h^{+}/O}^{2-}\to Intermediate\;products\to {CO}_{2}+H_{2}O$$

## 4. Conclusions

In this study, a novel Bi_2_WO_6_@GMs composite photocatalytic material was successfully prepared, and its adsorption–photocatalytic degradation performance for TC in water under visible light irradiation was systematically evaluated. Experimental results demonstrate that the system exhibits the optimal synergistic removal efficiency under the conditions of a 4% Bi_2_WO_6_ loading amount, a catalyst dosage of 1 g/L, and a weakly alkaline environment (pH 8). After 300 min of visible light irradiation, the degradation rate of TC reaches 82.7%. The material maintains structural stability and high-efficiency degradation capacity across tests in complex real-world water bodies (such as river water and tap water) as well as throughout four consecutive reuse cycles. Combined with scavenging experimental analysis, it was confirmed that photogenerated holes (h^+^) and superoxide radicals (O_2_^−^) generated from dissolved oxygen reduction are the core reactive species governing this highly efficient degradation process. Comparative analysis reveals that this composite material presents three significant advantages for practical antibiotic wastewater treatment:

(1) By utilizing widely available and low-cost geopolymer as the framework carrier, the usage of the relatively expensive active component Bi_2_WO_6_ is substantially compressed to a mere 4%. Furthermore, the entire synthesis and treatment process does not rely on costly precision instrumentation, drastically reducing raw material and processing costs.
(2) The preparation of the material requires neither stringent reaction conditions nor tedious multi-step chemical modifications, avoiding complex technological workflows and facilitating large-scale industrial production.
(3) The optimal working pH of the material perfectly matches natural water bodies, eliminating expensive acid–base pre-adjustment costs. Even in real natural water containing complex pollution matrices such as competing ions, microorganisms, and organic matter, it still maintains a considerable degradation rate.

In summary, the successful construction of the Bi_2_WO_6_@GMs composite system effectively resolves multiple obstacles faced by traditional powder photocatalytic systems regarding cost control, engineering scale-up, and adaptability to actual water quality. This research finding is expected to provide a potential reference for low-cost, large-scale practical environmental water remediation and industrial antibiotic wastewater treatment. However, further evaluation of the long-term stability and environmental safety of the material is still needed before practical application.

## Figures and Tables

**Table 1:** Kinetic parameters for TC degradation over different Bi_2_WO_6_@GMs samples.

Sample	Pseudo-First-Order		Pseudo-Second-Order	
	K_1_ (min^−1^)	R^2^	K_2_ (g·mg^−1^·min^−1^)	R^2^
2% Bi_2_WO_6_@GMs	0.0045	0.945	0.0021	0.993
4% Bi_2_WO_6_@GMs	0.0067	0.961	0.0031	0.998
6% Bi_2_WO_6_@GMs	0.0052	0.952	0.0024	0.995

**Table 2:** Comparison of Bi_2_WO_6_-based photocatalysts.

Photocatalyst	Mass Ratio		Synthesis Method	Initial Concentration	Catalyst Dosage	Reaction Conditions	Degradation Efficiency/Time	Ref
Bi_2_WO_6_	Bi_2_WO_6_ (100%)	/	Solvothermal method	TC (20 mg/L)	20 mg	LED lamp (90 W/m2), pH = 8	77.51%/160 min	[29]
I-Bi/Bi_2_WO_6_@MWCNTs	Bi_2_WO_6_ (93%)	multi-walled carbon nanotube (7%)	Solvothermal method	TC (20 mg/L)	20 mg	LED lamp (90 W/m2), pH = 8	96.75%/160 min	
Bi_2_WO_6_	Bi_2_WO_6_ (100%)	/	Hydrothermal method	TC (20 mg/L)	0.5 g/L	300 W Xe lamp (350 nm < λ < 780 nm)	50%/100 min	[30]
NiCo-LDH/Bi_2_WO_6_	Bi_2_WO_6_ (91%)	NiCo-LDH (9%)	Hydrothermal method	TC (20 mg/L)	0.5 g/L	300 W Xe lamp (350 nm < λ < 780 nm)	97%/100 min	
Bi_2_WO_6_	Bi_2_WO_6_ (100%)	/	Hydrothermal method	TC (25 mg/L)	0.3 g/L	Xe lamp, ultrasonication	47.1%/100 min	[31]
Cu_2_WS_4_/Bi_2_WO_6_	Bi_2_WO_6_ (95%)	Cu_2_WS_4_ (5%)	Two-step hydrothermal method	TC (25 mg/L)	0.3 g/L	Xe lamp, ultrasonication	80.0%/100 min	
Bi_2_WO_6_	Bi_2_WO_6_ (100%)	/	Hydrothermal method	TC (10 mg/L)	0.3 g/L	Visible light (300 W Xe lamp, λ > 420 nm)	79%/120 min	[32]
CuInS_2_/Bi_2_WO_6_	Bi_2_WO_6_ (90%)	CuInS_2_ (10%)	In situ hydrothermal method	TC (10 mg/L)	0.3 g/L	Visible light (300 W Xe lamp, λ > 420 nm)	92.4%/120 min	
BC/BWI	Bi_2_WO_6_ (56.8%)	BiOI (5.7%), Biochar (37.5%)	Solvothermal method	TC (50 mg/L)	0.1 g/L	Visible light (300 W Xe lamp, λ > 420 nm)	99.8%/60 min	[33]
Bi_2_WO_6_/BiOI@Fe_3_O_4_	Bi_2_WO_6_ (47.3%)	BiOI (47.7%), Fe_3_O_4_ (5%)	Hydrothermal method	TC (5 mg/L)	1 g/L	Visible light (500 W Xe lamp), pH = 7	97%/180 min	[34]
BiOCl/Bi_2_WO_6_	Bi_2_WO_6_ (80%)	BiOCl (20%)	Two-step hydrothermal method	TC (20 mg/L)	1 g/L	Visible light (350 W Xe lamp, λ > 420 nm)	94.0%/60 min	[35]
Bi_2_WO_6_: Er^3+^/*x*Yb^3+^	Bi_2_WO_6_ (97.78%)	Er and Yb (2.22%)	Solvothermal method, calcination	TC (20 mg/L)	1 g/L	Visible light (500 W Xe lamp, λ > 400 nm)	81.76%/30 min	[36]
N-CQDs/UBWO	Bi_2_WO_6_ (97%)	N-CQDs (3%)	In situ hydrothermal method	OTC (20 mg/L)	0.2 g/L	Visible light (300 W Xe lamp, λ > 420 nm)	85.0%/40 min	[37]
Bi_2_WO_6_/BiVO_4_	Bi_2_WO_6_ (10%)	BiVO_4_ (90%)	Two-step hydrothermal method, ultrasonication	OTC (20 mg/L)	0.1 g/L	Visible light (Xe lamp, λ > 400 nm)	96.7%/30 min	[38]
Bi_2_WO_6_@GMs	Bi_2_WO_6_ (4%)	Geopolymer (96%)	Calcination	TC (10 mg/L)	1 g/L	Visible light (300 W Xe lamp, λ > 400 nm), pH = 8	82.7%/300 min	This work

## Data Availability

The original contributions presented in this study are included in the article. Further inquiries can be directed to the corresponding author.
